# A randomised study evaluating the use of pyridoxine to avoid capecitabine dose modifications

**DOI:** 10.1038/bjc.2012.318

**Published:** 2012-07-19

**Authors:** P G Corrie, R Bulusu, C B Wilson, G Armstrong, S Bond, R Hardy, S Lao-Sirieix, D Parashar, A Ahmad, F Daniel, M Hill, G Wilson, C Blesing, A M Moody, K McAdam, M Osborne

**Affiliations:** 1 Oncology Division, Addenbrooke’s Hospital, Hills Road, Cambridge CB2 0QQ, UK; 2 Primrose Oncology Centre, Bedford Hospital, Bedford MK42 9DJ, UK; 3 Cambridge Cancer Trials Centre, Addenbrooke’s Hospital, Cambridge CB2 0QQ, UK; 4 Oncology Service, Queen Elizabeth Hospital, Kings Lynn, PE30 4ET, UK; 5 Plymouth Oncology Centre, Derriford Hospital, Plymouth PL6 8DH, UK; 6 Kent Oncology Centre, Maidstone Hospital, Maidstone ME16 9QQ, UK; 7 Department of Medical Oncology, Christie Hospital, Manchester M20 4BX, UK; 8 Oncology Service, Great Western Hospital, Marlborough Road, Swindon SN3 6BB, UK; 9 Department of Oncology, West Suffolk Hospital, Bury St Edmunds, IP33 2QZ, UK; 10 Haematology and Oncology Unit, Peterborough City Hospital, Peterborough PE3 9GZ, UK; 11 Exeter Oncology Centre, Royal Devon & Exeter Hospital, Exeter EX2 5DW, UK; 12 Cambridge Clinical Trials Unit, Addenbrooke’s Hospital, Cambridge CB2 0QQ, UK; 13 MRC Biostatistics Unit Hub in Trials Methodology Research, Forvie Site, Cambridge CB2 0SR, UK

**Keywords:** capecitabine, pyridoxine, hand–foot syndrome, randomised trial

## Abstract

**Background:**

Pyridoxine is frequently used to treat capecitabine-induced hand–foot syndrome (HFS), although the evidence of benefit is lacking. We performed a randomised placebo-controlled trial to determine whether pyridoxine could avoid the need for capecitabine dose modifications and improve outcomes.

**Methods:**

A total of 106 patients planned for palliative single-agent capecitabine (53 in each arm, 65%/ 35% colorectal/breast cancer) were randomised to receive either concomitant pyridoxine (50 mg po) or matching placebo three times daily.

**Results:**

Compared with placebo, pyridoxine use was associated with an increased rate of avoiding capecitabine dose modifications (37% *vs* 23%, relative risk 0.59, 95% CI 0.29, 1.20, *P*=0.15) and fewer grade 3/4 HFS-related adverse events (9% *vs* 17%, odds ratio 0.51, 95% CI 0.15–1.6, *P*=0.26). Use of pyridoxine did not improve response rate or progression-free survival.

**Conclusion:**

Pyridoxine may reduce the need for capecitabine dose modifications and the incidence of severe HFS, but does not impact on antitumour effect.

Hand–foot syndrome (HFS) is the most common adverse effect of capecitabine, with an incidence of 50–60% (Gressett *et al*, 2006), and its occurrence can lead to delay or discontinuation of treatment. Pyridoxine is frequently used to treat HFS, but the evidence of benefit is both limited and controversial (Beveridge *et al*, 1990; Wiernik *et al*, 1992; Chalermchai *et al*, 2010; Kang *et al*, 2010). We performed a randomised placebo-controlled trial to determine whether pyridoxine could reduce the incidence of capecitabine dose modifications.

## Materials and Methods

This multi-centre, randomised, double-blind, placebo-controlled NIHR portfolio phase 3 study was conducted at 10 UK sites. Patients with advanced colorectal or breast carcinoma receiving single agent capecitabine chemotherapy, ECOG performance status 0–2, life expectancy >12 weeks and aged >18 years gave written consent before entering the study, which had research ethics approval. Patients were randomised 1 : 1 and stratified by cancer site (colorectal or breast) to receive either pyridoxine (50 mg) or placebo orally three times daily commencing the same day that capecitabine chemotherapy was initiated. The capecitabine starting dose was planned to be 1250 mg m^−2^ administered orally, twice daily, for 2 weeks followed by 7 days rest. Treatment continued until disease progression, toxicity or patient preference. After discontinuation, patients were followed up for 12 weeks.

### Efficacy and safety evaluation

The primary endpoint of this study was incidence of capecitabine dose modifications at or before 12 weeks of treatment. Secondary endpoints were the incidence of HFS, quality of life (using the EORTC QLQ-C30 version 3 questionnaire including the modules dedicated specifically to colorectal and breast cancer), response to chemotherapy and progression-free survival (PFS). The incidence of HFS and other adverse events was recorded using the NCI CTCAE version 3.0 every 3 weeks before each chemotherapy cycle and for 12 weeks after completing the last cycle of chemotherapy. Quality of life was assessed before each cycle of chemotherapy and 12 weeks after stopping treatment. Tumour response was measured every 12 weeks using RECIST criteria. Progression-free survival was measured from the date of randomisation until the first date documented of disease progression.

### Statistical analysis

The study was designed to recruit 270 patients, to detect a reduction in the incidence of dose modification from 30 to 15% with 80% power and allowance for dropouts. The incidence of dose modifications was analysed using Kaplan–Meier curves and the ratio of modification-free rates at 12 weeks of treatment were estimated using 95% confidence intervals. Response rates at 12 weeks were analysed using logistic regression. Progression-free survival was analysed using Kaplan–Meier curves and the hazard ratio comparing the instantaneous rate of progression estimated. All randomised patients were included in the analyses on an intention-to-treat basis.

## Results

Between December 2004 and June 2009, 106 patients (53 in each arm) were recruited at 10 institutions in this NIHR portfolio trial. Slower than expected recruitment rate was due to decreasing numbers of patients being treated with capecitabine as a single agent and caused the study to close prematurely. Demographic comparison of both arms of the study identified no substantial imbalances, with median patient age 73 years, 64% female, 65% colorectal cancer and 93% performance status 0–1 (Table 1). Allowing for tablet strengths, 55% of patients commenced full-dose capecitabine, 42% started at a modified dose, whereas 3% of patients did not start their planned treatment. Nine patients were excluded from the analysis: three patients never commenced treatment and seven patients had missing data regarding dosing. The remaining 97 patients were included in the analysis: 49 in the pyridoxine arm and 48 in the placebo arm. The median number of weeks on study was 15 for both arms, approximating to five cycles of three weekly chemotherapy. At 12 weeks, 29 patients on the placebo arm and 37 patients on the pyridoxine arm remained on-study.

Table 2 summarises all grade 3 and 4 AEs reported in both arms of the study. The combined incidence of HFS-related AEs (‘HFS’ or ‘rash hand foot skin reaction’) showed a trend in favour of pyridoxine use: any grade was 51% *vs* 53% (odds ratio 0.93, 95% CI 0.43–2.0) whereas grade 3/4 was 9% *vs* 17% (odds ratio 0.51, 95% CI 0.15–1.6). There were no other differences in AEs between the two arms and no significant differences in quality of life. Summarising the Quality of Life QLQ-C30 using questions 29 and 30 (global health status and overall quality of life) observed on the second treatment cycle gave means (s.d.) of 66.1% (18.3%) and 62.5% (22.6%) for placebo (*n*=28) and pyridoxine (*n*=30), respectively; further exploratory analyses of different questions, subscales and time points revealed no differences.

**Figure 1:**
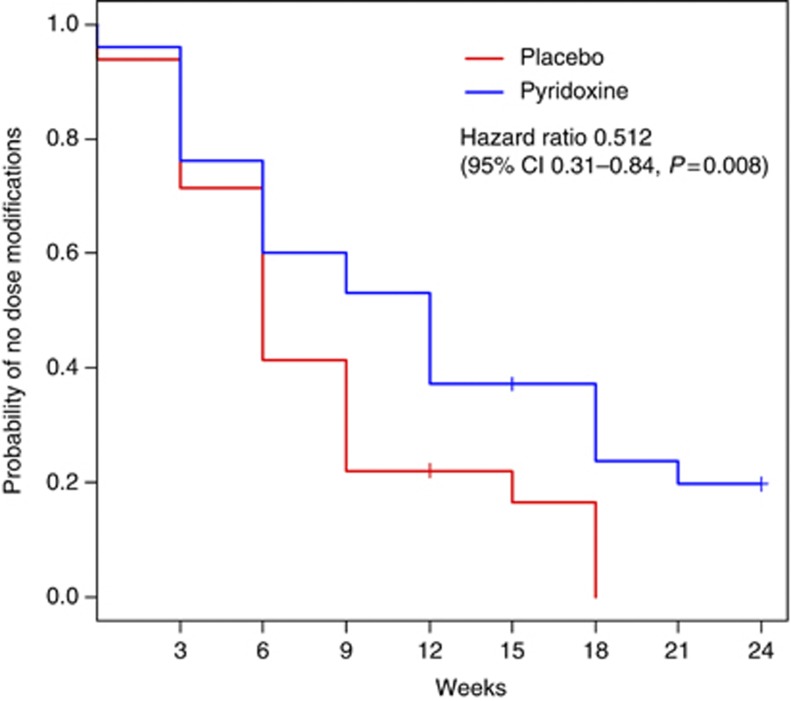
Kaplan-Meier estimates of the time to first dose modification.

At 12 weeks of treatment, the Kaplan–Meier estimate of remaining free from dose modification was 37% for patients receiving pyridoxine and 23% in patients receiving placebo (Figure 1); relative risk ratio 0.59 (placebo/pyridoxine, 95% CI 0.29, 1.20, *P*=0.152). Overall, pyridoxine had no significant effect on the objective response rate: 9 out of 46 responders on pyridoxine, and 9 out of 36 on placebo (placebo/pyridoxine odds ratio 1.37, 95% CI 0.475–3.96, *P*=0.56), but there was a trend towards pyridoxine decreasing PFS: median PFS durations of 7.4 months for pyridoxine and 9.9 months for placebo, hazard ratio 1.62 (pyridoxine/placebo 95% CI 0.91–2.88, log-rank *P*=0.095).

## Discussion

Although not life threatening, HFS may give cause for capecitabine dose delay, reduction or discontinuation, with a theoretical concern regarding compromise of potential overall efficacy of the planned chemotherapy. The pathogenesis of HFS is not known, but has been variously treated with emollients, steroids and non-steroidal agents (Gressett *et al*, 2006; Zhang *et al*, 2012). Because HFS resembles a rat disease, acrodynia, caused by pyridoxine deficiency, treatment with pyrodixine was proposed (Vukelja *et al*, 1989; Fabian *et al*, 1990). A small randomised study of 26 patients reported amelioration of HFS with pyridoxine 100 mg daily compared with placebo in patients receiving continuous infusional 5-Fluorouracil (Beveridge *et al*, 1990). Since then, two further randomised trials have been undertaken in SE Asian populations only. The first study of 389 patients reported that pyridoxine taken at 200 mg daily did not prevent HFS compared with placebo (Kang *et al*, 2010). The second smaller study of 56 patients compared 200 *vs* 400 mg concomitant pyridoxine and reported a trend in favour of improved HFS incidence and time to event with the higher pyridoxine dose (Chalermchai *et al*, 2010). In this UK study, 150 mg daily pyridoxine appeared to reduce the incidence of grade 3/4 HFS and the need for capecitabine dose modifications, although this did not translate into an improvement in outcome from chemotherapy itself: the trend towards poorer PFS in the pyridoxine arm was not statistically significant. It was not possible to detect any significant change in overall or specific elements of patient quality of life, using the QLQ-C30 instrument and the disease-specific modules.

Whether pyridoxine might in fact negatively influence chemotherapy efficacy is intriguing, although not conclusive. In their study of 200 *vs* 400 mg concomitant pyridoxine, Chalermchai *et al* (2010) reported significantly lower tumour responses to capecitabine at the higher pyridoxine dose level, whereas its use in advanced ovarian cancer was previously reported to reduce the duration of response to treatment with hexamethylamine plus cisplatin (Wiernik *et al*, 1992). Pyridoxine has in the past been considered an inexpensive method of avoiding the need to reduce capecitabine dosing. Pyridoxine has in the past been considered an inexpensive method of avoiding the need to reduce capecitabine dosing. Based on the CAPP-IT study results, its use as routine prophylaxis of HFS is not recommended.

## Figures and Tables

**Table 1:** Clinical characteristics

**Variable**	**Statistic**	**Placebo (** ***N*** **=53)**	**Pyridoxine (** ***N*** **=53)**
Age (years)	Mean (s.d.)	70 (10)	70 (9)
	Median	73	72
	Min, Max	42, 87	43, 84
			
Cancer type	Breast	34.0% (18/53)	37.7% (20/53)
	Colorectal	66.0% (35/53)	62.3% (33/53)
			
Performance status	1	85.7% (24/28)	88.0% (22/25)
	2	14.3% (4/28)	12.0% (3/25)
			
Gender	Male	37.7% (20/53)	37.7% (20/53)
	Female	62.3% (33/53)	62.3% (33/53)
			
Capecitabine	92–100%	54.7% (29/53)	56.6% (30/53)
starting dose	64–80%	41.5% (22/53)	41.5% (22/53)
(% full dose)	No treatment	3.8% (2/53)	1.9% (1/53)

**Table 2:** Summary of all grade 3 and 4 AEs reported

**AEs grade 3/4**	**Placebo (** ***N*** **=53) (%)**	**Pyridoxine (** ***N*** **=53) (%)**
HFS	9 (17)	5 (9)
Diarrhoea	6 (11)	1 (2)
Pain	2 (4)	3 (6)
Anorexia	2 (4)	0 (0)
Dyspnoea	2 (4)	0 (0)
Fatigue	1 (2)	2 (4)
Nausea	2 (2)	2 (4)

Abbreviations: AEs=adverse events; HFS=hand–foot syndrome.

## References

[bib1] Beveridge RA, Kales AN, Binder RA, Miller JA, Virts SG (1990) Pyridoxine (B6) and amelioration of hand/foot syndrome. Proc Am Soc Clin Oncol 9: 102a (abstr 393)

[bib2] Chalermchai T, Tantiphlachiva K, Suwanrusme H, Voravud N, Sriuranpong V (2010) Randomized trial of two different doses of pyridoxine in the prevention of capecitabine-associated palmar-plantar erythrodysesthesia. Asia-Pacific J Clin Oncol 6: 155–160 10.1111/j.1743-7563.2010.01311.x 20887495

[bib3] Fabian CJ, Molina R, Slavik M, Dahlberg S, Giri S, Stephens R (1990) Pyridoxine therapy for palmar-plantar erythrodysesthesia associated with continuous 5-fluorouracil infusion. Invest New Drugs 8: 57–63 2345070 10.1007/BF00216925

[bib4] Gressett SM, Stanford BL, Hardwicke F (2006) Management of hand-foot syndrome induced by capecitabine. J Oncol Pharm Pract 12: 131–141 17022868 10.1177/1078155206069242

[bib5] Kang YK, Lee SS, Yoon DH, Lee SY, Chun YJ, Kim MS, Ryu MH, Chang HM, Lee JL, Kim TW (2010) Pyridoxine is not effective to prevent hand-foot syndrome associated with capecitabine therapy: results of a randomized, double-blind, placebo-controlled study. J Clin Oncol 28: 3824–3829 20625131 10.1200/JCO.2010.29.1807

[bib6] Vukelja SJ, Lombardo FA, James WD, Weiss RB (1989) Pyridoxine for the palmar-plantar erythrodysesthesia syndrome. Ann Intern Med 111: 688–689 2529807 10.7326/0003-4819-111-8-688

[bib7] Wiernik PH, Yeap B, Vogl SE, Kaplan BH, Comis RL, Falkson G, Davis TE, Fazzini E, Cheuvart B, Horton J (1992) Hexamethylmelamine and low or moderate dose cisplatin with or without pyridoxine for treatment of advanced ovarian carcinoma: a study of the Eastern Cooperative Oncology Group. Cancer Invest 10: 1–9 10.3109/07357909209032783 1735009

[bib8] Zhang RX, Wu XJ, Wan DS, Lu ZH, Kong LH, Pan ZZ, Chen G (2012) Celecoxib can prevent capecitabine-related hand-foot syndrome in stage II and III colorectal cancer patients: result of a single-center, prospective randomized phase III trial. Annals Oncol 23: 1348–1353 10.1093/annonc/mdr400 21940785

